# Supplementary material for: A CRISPR-based assay for the study of eukaryotic DNA repair onboard the International Space Station
Source: PLoS One. 2021 Jun 30;16(6):e0253403. doi: 10.1371/journal.pone.0253403 (PMC8244870; doi:10.1371/journal.pone.0253403)
Supplement: S1 Video — Christina Koch first uses a pipette to dispense 120 μl of transformed Saccharomyces cerevisiae cells across the plate and then spreads them across the plate using a plastic spreader. (PDF) [file pone.0253403.s001.pdf]

S1 Video: https://twitter.com/Astro_Christina/status/1134184883297722368

Plating cells in microgravity. Christina Koch first uses a pipette to dispense 120 µl of transformed *Saccharomyces cerevisiae* cells across the plate and then spreads them across the plate using a plastic spreader.
