# Supplementary material for: A CRISPR-based assay for the study of eukaryotic DNA repair onboard the International Space Station
Source: PLoS One. 2021 Jun 30;16(6):e0253403. doi: 10.1371/journal.pone.0253403 (PMC8244870; doi:10.1371/journal.pone.0253403)
Supplement: S1 Fig — Colonies of interest were sequenced using Sanger methods and aligned to the expected ADE2 wild type (WT) sequence. Flight W2 was returned to the ground from the ISS and the colony re-streaked on a fresh plate prior to sequencing. Sanger sequencing data from this colony aligns to the wild type sequence as expected from the white colony phenotype. Sanger sequences show that Ground W1 contains a 9 bp deletion at position 52 and Ground W3 contains a 3 bp deletion at position 54. This supports nanopore sequencing data which showed low coverage at these positions. (PDF) [file pone.0253403.s002.pdf]

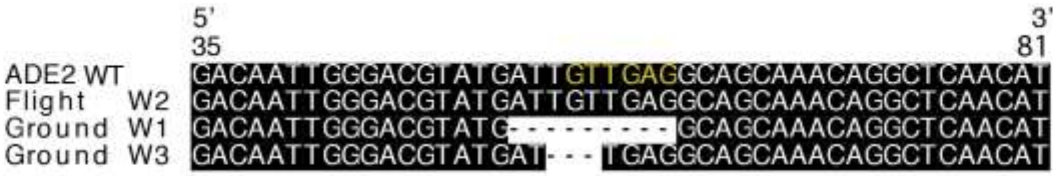

S1 Figure: Sanger sequencing confirmation of nanopore sequencing results. Colonies of interest were sequenced using Sanger methods and aligned to the expected *ADE2* wild type (WT) sequence. Flight W2 was returned to ground from the ISS and the colony re-streaked on a fresh plate prior to sequencing. Sanger sequencing data from this colony aligns to the wild type sequence as expected from the white colony phenotype. Sanger sequences show that Ground W1 contains a 9 bp deletion at position 52 and Ground W3 contains a 3 bp deletion at position 54. This supports nanopore sequencing data which showed low coverage at these positions.
