# Supplementary material for: A CRISPR-based assay for the study of eukaryotic DNA repair onboard the International Space Station
Source: PLoS One. 2021 Jun 30;16(6):e0253403. doi: 10.1371/journal.pone.0253403 (PMC8244870; doi:10.1371/journal.pone.0253403)
Supplement: S2 Fig — Nanopore reads are aligned to a hybrid reference sequence that contains both the ADE2 wild type sequence (white bars) and repair template sequence (red bars) at 43–80 bp. The bar graph depicts the number of reads at each base pair, thus lower coverage corresponds with a lower number of total reads per sample. A. Red colonies sequenced in flight show heterogeneity. 63.2%, 58.8%, 72.6%, and 75.1% of the reads from flight samples R1- R4 respectively map to the sequence of the repair template, while 49.3%, 55%, 35.4%, and 31.1% of reads mapped to the wild type sequence. In contrast, data from red colony ground controls R1-R4 show that 97.9%, 90.8%, 96%, and 94% of the reads map to the repair template sequence and 10.4%, 16.2%, 11.7%, and 13% reads mapped to wild type sequence. B. With the exception of Flight W2 and Ground W1 and W3, >98% of reads map to the wild type sequence from both flight and ground. Flight W2 observed similar mapping as red colonies with 76% reads mapping to the repair template sequence and 33% of reads mapping to the wild type sequence. Ground W1 and W3 had lower coverage mapped to wild type sequence due to the observed deletions (S1 Fig). (PDF) [file pone.0253403.s003.pdf]

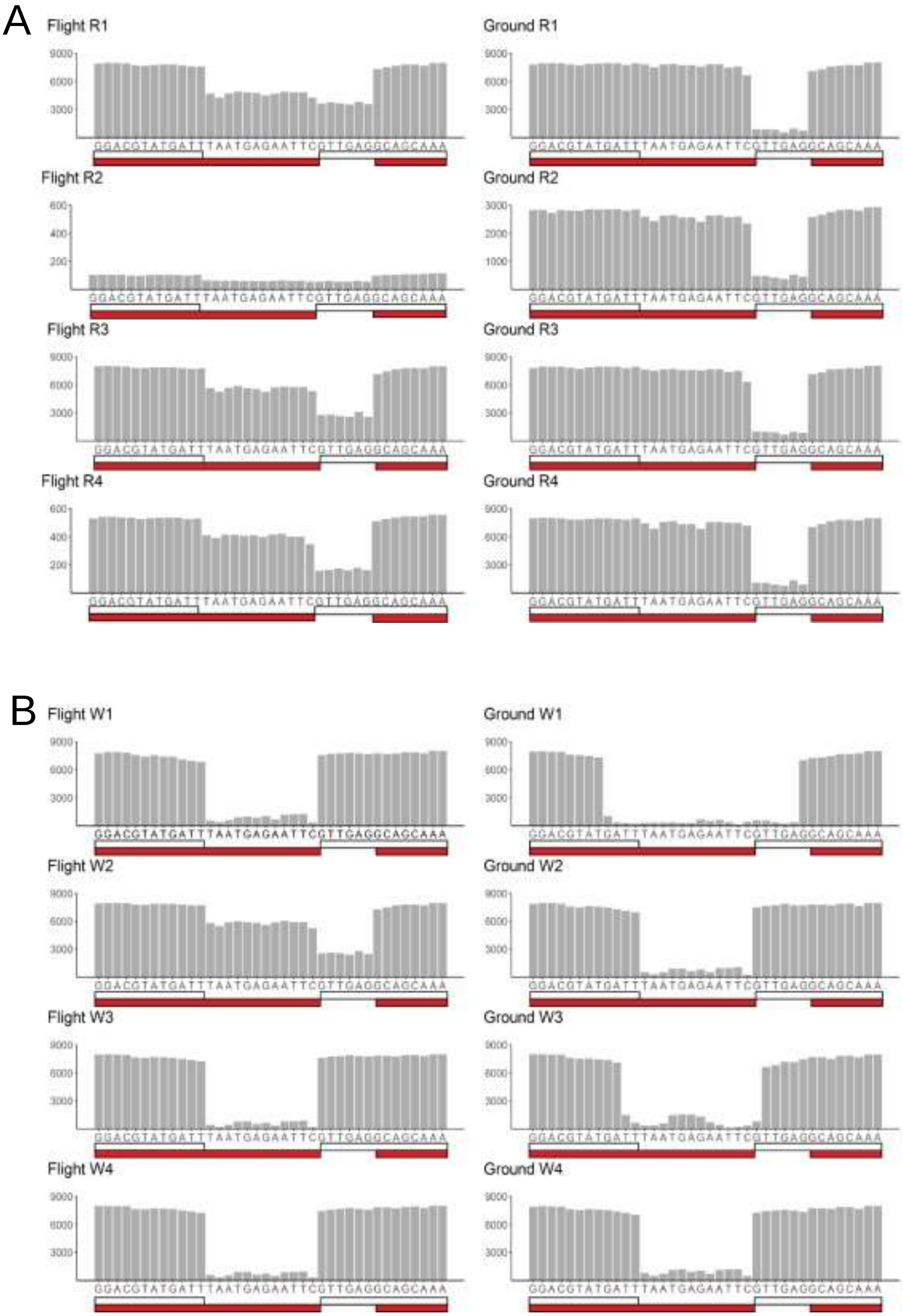
A
Flight R1
Ground R1
Flight R2
Ground R2
Flight R3
Ground R3
Flight R4
Ground R4
B
Flight W1
Ground W1
Flight W2
Ground W2
Flight W3
Ground W3
Flight W4
Ground W4
GGACGTATGATTTAATGAGAATTCGTTGAGGCAGCAAA

**S2 Figure:** Sequencing coverage plots of red and white colonies from flight and ground controls. Nanopore reads are aligned to a hybrid reference sequence that contains both the *ADE2* wild type sequence (white bars) and repair template sequence (red bars) at 43-80 bp. The bar graph depicts the number of reads at each base pair, thus lower coverage corresponds with lower number of total reads per sample. A. Red colonies sequenced in flight show heterogeneity. 63.2%, 58.8%, 72.6% and 75.1% of the reads from flight samples R1- R4 respectively map to the sequence of the repair template, while 49.3%, 55%, 35.4% and 31.1% of reads mapped to the wild type sequence. In contrast, data from red colony ground controls R1-R4 show that 97.9%, 90.8%, 96%, and 94% of the reads map to the repair template sequence and 10.4%, 16.2%, 11.7% and 13% reads mapped to wild type sequence. B. With the exception of Flight W2 and Ground W1 and W3, >98% of reads map to the wild type sequence from both flight and ground. Flight W2 observed similar mapping as red colonies with 76% reads mapping to the repair template sequence and 33% of reads mapping to the wild type sequence. Ground W1 and W3 had lower coverage mapped to wild type sequence due to the observed deletions (S1 Fig).
