# Supplementary material for: A CRISPR-based assay for the study of eukaryotic DNA repair onboard the International Space Station
Source: PLoS One. 2021 Jun 30;16(6):e0253403. doi: 10.1371/journal.pone.0253403 (PMC8244870; doi:10.1371/journal.pone.0253403)
Supplement: S1 Table — Includes total read count, demultiplexed read distribution, read length, and median alignment identity. A total of four nanopore sequencing runs were completed: pooled red colonies from flight, pooled white colonies from flight, pooled red colonies from ground, and pooled white colonies from ground. Reads were basecalled using Guppy and demultiplexed using Qcat. Read length determined with Samtools and Median Alignment Identity calculated from minimap2 alignments and Marginstats of red colonies to ade2 repair template reference and white colonies to ADE2 wild type reference. (PDF) [file pone.0253403.s004.pdf]

	Total Reads	Sample	Total Reads Per Sample	Reads Mapped	Read Length	Median Alignment Identity
Red Colony Flight Sequencing	583,026	R1	305,001	222,187	622.65	92.3%
		R2	8,543	393	520.43	91.8%
		R3	73,897	62,362	689.78	92.9%
		R4	775	635	683.66	92.6%
Red Colony Ground Sequencing	2,063,319	R1	1,021,290	826,906	638.84	92.8%
		R2	3,885	3,069	693.56	92.6%
		R3	603,619	515,223	689.12	93.1%
		R4	94,573	80,095	685.41	93.0%
White Colony Flight Sequencing	1,365,647	W1	858,631	703,191	654.92	92.3%
		W2	22,667	19,237	680.33	92.3%
		W3	136,095	116,656	687.88	92.7%
		W4	107,332	92,521	688.74	92.6%
White Colony Ground Sequencing	1,072,866	W1	510,586	428,699	656.48	90.8%
		W2	24,124	20,791	665.42	92.0%
		W3	76,803	65,354	672.35	91.6%
		W4	213,774	182,947	685.03	91.7%

S1 Table: Nanopore sequencing metrics including total read count, demultiplexed read distribution, read length, and median alignment identity. A total of four nanopore sequencing runs were completed: pooled red colonies from flight, pooled white colonies from flight, pooled red colonies from ground and pooled white colonies from ground. Reads were basecalled using Guppy and demultiplexed using Qcat. Read length determined with Samtools and Median Alignment Identity calculated from minimap2 alignments and Marginstats of red colonies to *ade2* repair template reference and white colonies to *ADE2* wild type reference.
