# Supplementary material for: A CRISPR-based assay for the study of eukaryotic DNA repair onboard the International Space Station
Source: PLoS One. 2021 Jun 30;16(6):e0253403. doi: 10.1371/journal.pone.0253403 (PMC8244870; doi:10.1371/journal.pone.0253403)
Supplement: S2 Table — AMP277 and AMP278 primers flank the Cas9 cut site specified by the ADE2 guide RNA and contain adaptors for the LWB barcodes used in this study. AMP306 and AMP308 were used for Sanger sequencing confirmation of select colonies upon return to ground. (PDF) [file pone.0253403.s005.pdf]

Primer	Sequence
AMP277	5’ ACTTGCCTGTCGCTCTATCTTCTGAACGGAGTCCGGAACTCTAGCAG 3’
AMP278	5’ TTTCTGTTGGTGCTGATATTGCCTAGAACAGTTGGTATATTAGGAGG 3’
AMP306	5’ TGTATAAATTGGTGCGTAAAATCGTTGG 3’
AMP308	5’ CATCAACATGCTCAATCTCAATCG 3’

S2 Table: Primers used in this experiment. AMP277 and AMP278 primers flank the Cas9 cut site specified by the *ADE2* guide RNA and contain adaptors for the LWB barcodes used in this study. AMP306 and AMP308 were used for Sanger sequencing confirmation of select colonies upon return to ground.
