# Supplementary material for: A CRISPR-based assay for the study of eukaryotic DNA repair onboard the International Space Station
Source: PLoS One. 2021 Jun 30;16(6):e0253403. doi: 10.1371/journal.pone.0253403 (PMC8244870; doi:10.1371/journal.pone.0253403)
Supplement: S3 Table — Sanger sequencing was used to verify Nanopore sequencing results for Ground W1, Ground W3, and Flight W2. (PDF) [file pone.0253403.s006.pdf]

**Ground W1**	TTGTATAAATTGGTGCGTAAAATCGTTGGATCTCTCTTCTA<br>AGTACATCCTACTATAACAATCAAGAAAAACAAGAAAATCG<br>GACAAAACAATCAAGTATGGATTCTAGAACAGTTGGTATAT<br>TAGGAGGGGGACAATTGGGACGTATGGCAGCAAACAGGCT<br>CAACATTAAGACGGTAATACTAGATGCTGAAAATTCTCCTGC<br>CAAACAAATAAGCAACTCCAATGACCACGTTAATGGCTCCTT<br>TTCCAATCCTCTTGATATCGAAAAACTAGCTGAAAAATGTGAT<br>GTGCTAACGATTGAGATTGAGCATGTTGATGN
**Ground W3**	TTGTATAAATTGGTGCGTAAAATCGTTGGATCTCTCTTCTAAGT<br>ACATCCTACTATAACAATCAAGAAAAACAAGAAAATCGGACAAA<br>ACAATCAAGTATGGATTCTAGAACAGTTGGTATATTAGGAGGGG<br>GACAATTGGGACGTATGATTGAGGCAGCAAACAGGCTCAACATT<br>AAGACGGTAATACTAGATGCTGAAAATTCTCCTGCCAAACAAATA<br>AGCAACTCCAATGACCACGTTAATGGCTCCTTTTCCAATCCTCTT<br>GATATCGAAAAACTAGCTGAAAAATGTGATGTGCTAACGATTGAG<br>ATTGAGCATGTTGATGA
**Flight W2**	AAATTGGTGCGTAAAATCGTTGGATCTCTCTTCTAAGTACATCCTA<br>CTATAACAATCAAGAAAAACAAGAAAATCGGACAAAACAATCAAGT<br>ATGGATTCTAGAACAGTTGGTATATTAGGAGGGGGACAATTGGGA<br>CGTATGATTGTTGAGGCAGCAAACAGGCTCAACATTAAGACGGTA<br>ATACTAGATGCTGAAAATTCTCCTGCCAAACAAATAAGCAACTCCA<br>ATGACCACGTTAATGGCTCCTNNNCAATCCTCTTGATATCGAAAAA<br>CT

S3 Table: Sanger sequencing data. Sanger sequencing was used to verify Nanopore sequencing results for Ground W1, Ground W3, and Flight W2.
